# School engagement and implications for future opportunities: Roma students’ trajectories

**DOI:** 10.3389/fpsyg.2025.1535063

**Published:** 2025-09-18

**Authors:** Tânia Moreira, Pedro Rosário, Márcia Ferreira, Armanda Pereira, Sonia Fuentes

**Affiliations:** ^1^Research Center in Psychology, School of Psychology, University of Minho, Braga, Portugal; ^2^Department of Education and Psychology, University of Trás-os-Montes and Alto Douro, Vila Real, Portugal; ^3^Faculty of Education, Central University of Chile, Santiago, Chile

**Keywords:** school engagement, Roma education, elementary education, educational trajectories, future opportunities, educational inequality, qualitative longitudinal research

## Abstract

**Introduction:**

Roma students often face challenges such as discrimination and social exclusion that hinder their school engagement. However, little is known about how their engagement changes over time and how this affects future life opportunities.

**Methods:**

This qualitative, longitudinal study followed 13 Roma students (ages 10–14) during a critical school transition (5th to 6th grade). Semi-structured interviews were conducted at the beginning and end of the school year and analyzed thematically.

**Results:**

Findings revealed that students maintained strong connections with peers and the school environment but showed weaker engagement in classroom learning. Over time, declining teacher relationships, reduced attendance, and limited academic support at home further diminished engagement. These patterns were linked to a low perceived value of formal education and restricted aspirations shaped by cultural norms.

**Discussion:**

The study highlights how social, familial, and cultural factors intersect to influence Roma students’ engagement trajectories. Interventions are needed that both build on cultural strengths and address motivational beliefs and structural barriers to enhance long-term educational opportunities.

## Introduction

Roma is the most significant ethnic minority in Europe and one of the most deprived ethnic groups, encountering similar challenges in all European countries, such as poverty, inadequate housing, underemployment, and underperformance in education (Council of Europe, 2020a). Education has been targeted as a critical hub for addressing poverty and ensuring equal opportunities in vital areas like employment and healthcare (Council of Europe, 2020b; Hamilton, 2018; Rosário et al., 2020). Nonetheless, students from Roma groups often face additional challenges that may hinder their access to and success in formal education (Moreira et al., 2022a, 2023a). Despite some improvements in literacy rates (Rutigliano, 2020), children from Roma communities still lag far behind their non-Roma counterparts in developmental tasks such as school engagement and achievement (Direção-Geral de Estatísticas da Educação e Ciência [DGEEC], 2019; Council of Europe, 2020b; European Union Agency for Fundamental Rights (FRA), 2021; Frazer and Marlier, 2011). Within the national context, data for Roma groups indicate a considerable increase in overall school achievement between the 2016/17 and 2018/19 school years, with elementary school rates rising from 56 to 76.4% and high school rates increasing from 64 to 75.4% (Direção-Geral de Estatísticas da Educação e Ciência [DGEEC], 2019). However, this progress is juxtaposed against an alarming rise in dropout rates. In elementary schools, the national dropout rate increased from 5.9% (2016/17) to 8% (2018/19), while in high schools, it went up from 5.6% (2016/17) to 11.9% (2018/19) (Direção-Geral de Estatísticas da Educação e Ciência [DGEEC], 2019). Notably, just approximately 3% of young people from Roma groups attend high school (Direção-Geral de Estatísticas da Educação e Ciência [DGEEC], 2019; Moreira et al., 2023a), and 6% of the youth from Roma groups aged between 16 and 24 are not enrolled in any education, employment, or training.

To tackle these educational disparities, research should address the mechanisms underlying the school (dis)engagement and integration of students from Roma groups. School Engagement (SE) is well documented as a crucial predictor of students’ school achievement (Chase et al., 2014), reduced instances of behavioral problems (Hirschfield and Gasper, 2010; Li and Lerner, 2011), and decreased risk of dropping out of school (Fall and Roberts, 2012). However, children from Roma groups navigating between their heritage and mainstream cultural references face unique challenges likely to impact their sense of belonging and success (Kraus, 2012; Wilding, 2008). The literature presents conflicting reasons for the disengagement among Roma students. Some authors (e.g., Lauritzen and Nodeland, 2018) stress cultural dissonance and lack of interest as primary causes of this disengagement, while others (e.g., Hamilton, 2018; Želinský et al., 2021) find these reasons overly simplistic and a pivotal hub to perpetuate exclusion (Caetano et al., 2024; Lauritzen and Nodeland, 2018). Moreover, these explanations fail to reflect the dynamic and context-dependent nature of (dis)engagement. Despite the predictive power of school engagement to academic success and future life opportunities, existing research has largely overlooked how (dis)engagement unfolds for students from Roma groups (Rosário et al., 2016, 2017). Few studies have examined (dis)engagement within Roma communities from a longitudinal perspective to investigate how these developmental tasks fluctuate as students move from the beginning to the end of the school year, influenced by their relational environment in school. Moreover, the voices of students from Roma groups regarding their own educational experiences and perceptions remain largely underrepresented in the literature (Caetano et al., 2024). To fill this literature gap, the current study followed a two-wave qualitative approach to investigate the (dis)engagement trajectories of 5th and 6th-grade students from Roma groups over a school year. We aim to gain insights into the underlying reasons for the persistent underachievement and school dropout among students of Roma backgrounds while also contributing to reforming policies and initiatives to enhance the educational engagement of Roma communities.

## Theoretical framework

### Acculturation and school engagement

Schools are privileged contexts for the developmental and acculturation processes for marginalized ethnic groups such as Roma (Makarova, 2019; Mendes et al., 2023; Ward and Geeraert, 2016). Acculturation is a dynamic process unfolding in the continuous contact between two or more cultural groups, influencing behaviors, values, and identities (Berry, 1997; Berry, 2005; Makarova and Birman, 2015). Within educational settings, this process is shaped by both micro-level interactions (e.g., peer and teacher relationships) and macro-level structures (e.g., systemic discrimination, curriculum relevance; Ward and Geeraert, 2016). School engagement is both an outcome, a driver of acculturation, and a predictor of future life prospects and adaptation (Fredricks et al., 2016; Schachner et al., 2017; Motti-Stefanidi, 2023). Conversely, disengagement is related to underachievement, poor health and psychological outcomes, low opportunities in the labor market, and increased risk of deviant behaviors (e.g., delinquency and substance use) (e.g., Hancock and Zubrick, 2015; Wang and Fredricks, 2013; Wang et al., 2019). Despite lacking consensus in the literature, engagement and disengagement can be described as opposite states of the same continuum, with disengagement reflecting a lack of engagement (Fredricks et al., 2016, 2019; Wang and Degol, 2014).

School engagement refers to students’ active participation and involvement in school activities, including classes, projects, and extracurricular activities. It is a complex construct that seeks to understand how students feel, act, and think (Azevedo et al., 2023). According to Fredricks et al. (2004), school engagement encompasses three interrelated dimensions: emotional, behavioral, and cognitive. The emotional dimension refers to students’ positive feelings toward people in the school community, as well as students’ emotional bond with the school, including the sense of belonging and enjoyment. The behavioral dimension includes the active participation of students in school activities, such as attending classes, doing homework, and participating in extracurricular activities. Finally, the cognitive dimension refers to the student’s attention, interest, and effort in learning, encompassing a set of cognitive strategies used to improve their learning processes.

From a social-cognitive perspective, school engagement is shaped by students’ perceptions of value, belonging, and the cultural relevance of their educational experiences. According to expectancy-value theory (Eccles and Wigfield, 2002), students are more likely to engage when they perceive school as valuable and attainable. Research shows that emphasizing the utility value of school can enhance motivation and academic outcomes, especially among underrepresented students (Harackiewicz et al., 2016). When students recognize the relevance of education to their future goals, they are more likely to persist and aspire to higher education (Guo et al., 2015). However, students from Roma groups frequently face systemic barriers (e.g., low teacher expectations, pressure to assimilate, and limited access to high-quality education) that undermine both their expectancy of success and the perceived utility of education, leading to disengagement and reduced participation in mainstream educational and social systems (Caetano et al., 2024; Motti-Stefanidi, 2018; Rosário et al., 2013a,b; Mendes et al., 2023; Ward and Geeraert, 2016). Moreover, the lack of cultural capital within families can further hinder engagement by limiting parental support and reinforcing feelings of exclusion. Despite increasingly supporting education, mothers from Roma groups often aspire to secure basic needs for their children rather than pursue upward class mobility (Berki et al., 2025; Moreira et al., 2022b, 2023b).

While school engagement is widely recognized as a key predictor of academic success and long-term life opportunities (Fredricks et al., 2004; Schachner et al., 2017), there is a significant gap in understanding how engagement unfolds over time for Roma students, particularly during critical educational transitions. Most existing studies offer cross-sectional snapshots or focus on structural barriers without capturing the dynamic, context-sensitive nature of engagement and disengagement processes. Therefore, by uncovering the mechanisms that sustain or erode engagement over time, this study contributes to a more comprehensive understanding of educational pathways of students from Roma groups. It also provides actionable insights to design culturally responsive interventions that foster inclusion and long-term success for youth from minoritized communities.

### The present study

Despite the considerable efforts made by European policies and research to favor successful school transitions of students with Roma backgrounds beyond compulsory education, the outcomes are modest (e.g., high early dropout and absenteeism rates and persistent high underachievement) (Caetano et al., 2024; Mendes and Magano, 2021). Addressing students’ engagement in education is crucial for young individuals to fully benefit from the educational experience and develop the necessary skills to thrive in today’s competitive world (Azevedo et al., 2023). For example, Stenroos and Helakorpi (2021) state that exploring the motivational drivers for students with Roma backgrounds to attend and pursue school beyond elementary education is crucial. Grounded in acculturation and school engagement frameworks, and employing a multiple-case study design, this study examines the (dis)engagement patterns of 5th- and 6th-grade students with Roma backgrounds over a school year. The 5th and 6th grades were selected because they represent crucial school transitions in the Portuguese educational system (from elementary to middle school). During this transition, students face changes related to the teaching methods, curriculum content, academic expectations, and social dynamics, which are likely to influence their (dis)engagement and achievement.

Furthermore, at this stage, students from Roma communities have already acquired fundamental literacy and numeracy skills that are highly valued within the Roma community for their practical utility (e.g., getting a driving license, preparing to marry, or working in a fair selling clothing). Consequently, this foundational achievement may diminish their perceived need for further education, undermining their motivation to pursue additional learning opportunities. Altogether, findings are expected to build a more nuanced portrait of the developmental tasks (i.e., school engagement) and provide insights into the underlying mechanisms supporting the three dimensions of engagement at the end of elementary and transition to middle school. Moreover, data can help to identify potential barriers and challenges these students face, which is helpful in the development of targeted interventions and support systems to enhance their school experiences.

### Context

To help capture different school environments and students’ cultural dissimilarities, this study was conducted in two purposefully selected public schools located in underprivileged areas within two cities in northern Portugal. These schools were chosen because of the large and diverse population of students from Roma groups. Despite being perceived as homogeneous, Roma groups in Portugal differ in their cultural traditions and values, economic activities, and housing. The two selected schools include families from three main Roma communities: the first group comprises people living in social housing, who generally sell clothes at fairs; the second group includes individuals also settled in social housing who typically sell toys and balloons at traditional and popular fairs; while the third group comprises individuals living in tents with even more limited resources than those of the first two groups. Individuals in the third group engage in economic activities related to subsistence agriculture and selling scrap metal.

## Method

### Sample

To ensure diversity within the target population, participants were recruited using an intentional sampling strategy (Robinson, 2014), guided by the following inclusion criteria: (a) school identification as belonging to a Roma ethnic group, (b) enrollment in the 5th or 6th grade in one of two selected public schools, and (c) availability for participation in both waves of data collection. The sample was drawn from two public schools in socioeconomically disadvantaged areas with a high concentration of Roma students. The sample included students from three distinct Roma subgroups, each with different socioeconomic and cultural characteristics (i) families living in social housing and primarily engaged in clothing sales at fairs; (ii) families also in social housing but involved in selling toys and balloons at traditional fairs; and (iii) families living in tents or informal settlements, often engaged in subsistence agriculture or scrap metal collection. Seventeen students participated in the first wave of interviews. Four were lost to follow-up due to relocation or health issues. For the present study, only those participants who completed interviews in both waves were considered for the analysis. The final sample consisted of 13 students (8 in 5th grade, 5 in 6th grade), aged 10–14 years, with five identifying as female. Approximately 69% had experienced grade retention during their trajectory.

### Procedures

The research was conducted upon approval by the Scientific Ethics Committee of the University of Minho and the Portuguese Ministry of Education. Recruitment was facilitated through school directors, teachers, and social workers, who helped establish trust with families. Parents or legal guardians were invited to an individual in-person meeting at school, where the study was explained in accessible language. Written informed consent and student assent were obtained. It was emphasized that their involvement was completely voluntary and confidential, and they had the right to withdraw from the study at any point without any consequences. No incentives or financial compensation were provided to the participants. Two experienced investigators conducted the interviews in both groups. To minimize power imbalances and cultural misunderstandings, interviews were conducted by two experienced researchers with prior familiarity with Roma communities.

### Data collection

Semi-structured interviews were conducted at two time points of the school year (At the beginning, M1 -September, and at the end of the same school year, M2 – June). Interviews were held in quiet, familiar school settings and began with rapport-building conversation to reduce cultural and linguistic barriers (Lyberg et al., 2014). Open-ended questions explored emotional, behavioral, and cognitive dimensions of school engagement. Sample questions include: “What do you like the most at school? What do you like the least?,” “How often do you come to school?, What are the reasons for being absent from school?,” “How important is the school in your life? How can the school help you in your future?.” The second-wave interviews included reflective prompts to capture perceived changes over the year. For example, “Another school year has come to an end. How was this school year for you? [How were your grades? Your behavior?]”; “During this year, have you thought about something you would like to do when you grow up a little more? How do you think your life will be?”; “Until when do you think you will continue to be enrolled in school? Why?.” All participants were proficient in Portuguese, the language of instruction in their schools. However, recognizing the potential for linguistic nuances and expression gaps, interviewers deliberately tried to ensure comprehension. This included rephrasing questions, using culturally familiar terms, and checking for understanding throughout the conversation. Each interview lasted between 30 and 45 min and was recorded in audio and transcribed verbatim, guaranteeing anonymity and confidentiality.

### Data analysis

The analysis sought to identify themes and patterns of school engagement trajectories and the inner workings of those trajectories over a school year. Thematic content analysis was helped by the NVivo software, following the recursive process by Braun and Clarke (2012). A hybrid coding strategy was used in the codebook development. Deductive codes were derived from the theoretical framework (e.g., emotional, behavioral, cognitive engagement), while inductive codes emerged from the data. Intra- and cross-case analyzes were performed, using a constant comparative method to map possible relationships across perspectives and time points. Demographic characteristics (e.g., subgroup affiliation, gender, grade level) were considered in the analysis to capture potential influences on school engagement trajectories. However, due to sample size limitations, some variables (e.g., subgroup affiliation) were not used as a primary axis of comparison. To enhance analytic rigor and mitigate bias, two authors have coded more than 20% of the data (*n* = 6) independently to ensure coding consistency. Cohen’s kappa ranged from 0.80 to 0.99, suggesting almost perfect consistency between coders. Discrepancies were resolved through discussion and consensus, and the codebook was refined accordingly. Regular peer debriefing sessions were held to challenge interpretations and ensure alignment with participants’ narratives. Theme frequencies were reported using the scheme proposed by Cooper and Rodgers (2006). The term “all” is used to describe 100% of participants, “nearly all” to refer to 100% − 2 participants, “most” to describe a range between 50% + 1 to 100% − 2, “around half” to describe 50% + 1 participants, “some” to refer to a range between 3 and 50% + 1 participants, “a couple” to indicate 2 participants, and “one” to describe one participant (see Table 1). Supporting quotes were included to illustrate themes and subthemes, provide a detailed description of participants’ representations and experiences, and add validity to the findings (Smith, 2017).

**Table 1:** Frequency of responses.

Frequency	Score scheme	Number of participants (*N* = 13)
All	100%	12–13
Nearly all	100% − 2	11
Most	50% + 1 to 100% − 2	8–10
Around half	50% + 1	6–7
Some	3 to 50% + 1	3–5
A couple	2	2
One	1	1

### Findings

Results are presented in two sections: (I) school engagement trajectories and (II) Motivational Drivers of School Engagement.

#### Section I: school engagement trajectories

Indicators of school engagement were assessed regarding its three dimensions: (a) emotional, (b) behavioral, and (c) cognitive engagement. Data were compared across timepoints (M1 and M2) and school grades to examine whether findings changed over time and across school grades. Differences between the two waves will be reported when they exist. No meaningful differences were found in the comparisons across school grades, even when accounting for gender or age.

##### Trajectories of emotional engagement

The emotional dimension encompasses feelings toward school, such as enjoyment or a sense of belonging, which are shaped by the school environment and interactions with classmates and teachers. Overall, students showed mixed feelings about school. There was a slight increase in positive emotions reported toward the school environment during non-curricular activities (e.g., school breaks) from M1 to M2. In contrast, participants consistently expressed negative emotions toward structured learning activities (i.e., classroom environment) at both timepoints.

Ah, [I like the school] when there are breaks, when I do not have classes, when the teacher is absent and gets substituted (SL01).

I like the school, but I do not like the classes…I do not like to study or do the homework. I do not like it when the teacher is explaining and I must listen to her… I do not like it (DL02).

This dual trajectory of engagement seems to be linked to the relationships and interactions established in each context. Participants primarily enjoyed non-academic activities within their Roma peer groups. Participants primarily enjoyed non-academic activities within their Roma peer groups. All participants reported positive relationships with their Roma peers, identifying them as the primary source of social support and belongingness. Conversely, classroom settings more often involved interactions with non-Roma peers. A consistent lack of positive cross-cultural peer relationships was observed. Instances of inter-ethnic conflicts, such as name-calling and physical aggression, were frequent at the beginning of the year. By the end of the year, there was a slight decrease in the reported conflicts, but they were still present for almost half of the students.

Yes, I like the playgrounds and hanging out with my friends. Most of the time, I am with the other Roma [LR01].

[Who are the people at school with whom you get along better?] My cousins. [Who are your cousins?] The Roma. [Are they all your cousins?] Yes (MR02).

Regarding classroom learning, the number of participants reporting positive teacher-student relationships decreased over time. While half the participants reported positive relationships with specific teachers at both time points, this was not a generalized experience across all teachers.

[And how about the teachers?] I like the English teacher. [Why?] She helps me get better grades. [How does she help you?] She pushes me. (…) Sometimes, I’m distracted, and the teacher helps me focus more. I am now more concentrated in class. But there are others… The Math one. (…) She always scolds. (…) Everyone in class. (…) We go in, and she’s already there, and she says, “All right! All right, you bunch of idiots”. (…) She says things like that. [And what do you think of that?] It’s bad. [And does that always happen?] Yes (JN01).

We are over there (…) and she’s already fidgeting and calling us names. “I don’t want donkeys, I don’t want dummies here, I don’t want a pile of straw here, donkeys, what are they for here” she says something that scares me. That’s why I don’t like her and sometimes I’m like, “Yeah? What am I going to work for? It’s not worth it”. I turn away, she speaks and I ignore her. That’s why I don’t make an effort in math because of the teacher, she doesn’t explain anything (MA02).

##### Trajectories of behavioral engagement

The behavioral dimension reflects the idea of participation and involvement in academic and social or extracurricular activities. At the beginning of the year, most participants reported regular attendance. However, by the end of the year, most participants reported irregular attendance. The reasons for frequent absences ranged from attending cultural obligations (e.g., visiting relatives at the hospital or helping parents at fairs) to low motivation. This downward trend in attendance and punctuality is consistent with decreased commitment to academic tasks (e.g., completing homework) and a rise in maladaptive behaviors.

[skip school] I use to skip some classes…because I’m sick, (…) or because I fall asleep or don’t feel like it or because I made things up (LR01).

I have around 104 absences… Most of them in the beginning… Many things happened, like my grandma broke her arm and I stayed at home… [Why?] I don’t know… I stay there playing, playing on the tablet (LR02).

[Do you usually study outside of school?] I study at the Study Center [activities developed by an NGO]. [And do you only do homework or study too?] We do homework and study. Then, in the end, we go to the computers. [When you don’t go to the study center, do you usually study at home or do homework at home?] Yes (ID01).

[What about homework?] I never did. [Why] Because I was playing instead (ID02).

Despite this disengagement from learning aspects, participation in non-curricular activities (e.g., playing soccer, playing on computers) followed a stable and positive trajectory throughout the school year. All participants reported behavioral engagement in social and recreational activities.

I play soccer, or I play on computers with them (ID01).

I’m always playing with my peers (DL02).

##### Trajectories of cognitive engagement

The cognitive dimension encompasses the notion of commitment, involving reflection and willingness to exert effort in understanding intricate concepts and developing challenging skills. Overall, data suggest a surface-level cognitive engagement among participants. There was a downward trend in their efforts to seek help, plan and organize, and self-evaluate. Over the school year, the number of participants who reported having support in their learning tasks decreased from most in M1 to one in M2. The predominant explanation cited by participants related to family members’ low literacy, which hindered their ability and openness to provide support. Seeking help from teachers was mentioned by some participants at the beginning of the school year, decreasing to one by the end of the school year.

Because I sometimes ask my mother, and my mother doesn’t know [not able to explain the school content] because she only studied until the sixth grade (DL01).

Regarding other cognitive strategies, some participants demonstrated an awareness of steps needed for academic tasks. However, the occurrence of these strategies was limited.

Oh, I put the book and the notebook, I put a sheet next to it. And I see what’s in the book and the notebook, I go to previous classes… And I see what’s written there. Then I take a sheet and write what’s there (SL01).

I go to the notebook to see what I have [timetable]: math. Sometimes, I have a test, and I have to study a little more. I study for an hour, an hour and a half, make revisions, close the book, check if it’s right, do the exercises, and that’s it. I save it, do my homework, and put it in the book. Then I wake up in the morning, my mother makes me a snack, and I put it in my backpack. And I go to school (MA02).

All participants engaged in judgments related to their functioning and behaviors regarding learning processes at both time points. This awareness covered recognizing issues like talking too much in class or neglecting homework and acknowledging their abilities and weaknesses. However, despite being stable over time, this awareness seems not to have translated into adaptive strategies to improve. As illustrated by the following quotes, there is a potential gap between students’ recognition of what they should change and their ability to enact behavioral strategies likely to help them overcome difficulties or limitations.

Because I don’t study. (…) I’m talking a lot in class. I never do homework. I never study (ID02).

Why is it important to be smart? (…) To always get 100% on the tests, I wish. There’s a girl who always does it; she’s always in the nineties [percentage] or in the hundred [percentage]. [She is] Smarter. Intelligent. [What do you need to be more intelligent and smarter like that?] Study, pay attention (AM02).

#### Section II: motivational drivers of school engagement trajectories

For this group, perceptions of the *utility value of education* emerged as an underlying driver of their engagement trajectories in school and classroom environments. Utility value beliefs refer to education’s perceived importance and practical benefits to students’ lives. These beliefs are coded into four key codes: (1) mastering literacy and numeracy skills as gateways to independence; (2) opening future life opportunities in the labor market; (3) improving social and cultural capital; and (4) avoiding adverse outcomes.

Across the school year, all participants acknowledged the immediate value of education for mastering basic skills, such as literacy and numeracy, portrayed as gateways to independence in daily tasks such as reading official documents, signing contracts, managing finances, and getting a driver’s license.

In a few years, I learned how to read and how to explain things to my children. Knowing how to read. Because I started reading at school, I started writing at school, I think that’s it (NZ01).

Know how to read and write to get a driver’s license. To teach my parents (ID01).

Additionally, a slight upward trend was noticed in the long-term benefits of education, with students portraying education as a tool to secure future life opportunities, such as paid jobs. By the end of the year, nearly all participants viewed education as a pathway to job opportunities and financial stability.

It is for us to study, then to have a job. A future. Because it helps us to have a future (DL01).

This contrasts with a downward trend in viewing school as a source of social and cultural capital development. At the beginning of the school year, half of the participants referred to education as a tool to acquire social skills, but by the end of the school year, only some students mentioned this. Moreover, at the end of the school year, most participants focused their motivations to attend school on avoiding governmental sanctions, such as losing social allowances or being placed in residential care institutions.

To learn to read, and to know how to be. [How to be? With other people?] Yes. With friends. Learn to speak too. [Do you think the school teaches how to speak?] Not to speak… but to talk to people (SL01).

[How long do you think you’ll be in school for?] I don’t know. If I behave well, I’ll stay until the end. [And when does school end?] I don’t know. [You must go to school until when?] Seventeen, right? [It’s mandatory until you’re eighteen] Eighteen. [Will you go to school until you’re required to?] Yes. [Why do you think they make you go to school?] Because it’s the law (NZ02).

Overall, findings suggest that students’ perceived utility value of education relies on instrumental purposes (e.g., getting a paid job, avoiding family disruption, or fulfilling legal requirements) rather than valuing learning for its intrinsic benefits (e.g., personal growth). This instrumental lens may contribute to decreased emotional, behavioral, and cognitive engagement with learning activities over time.

## Discussion

This study was grounded on the theoretical connection between school engagement, achievement, and future long-term adaptation (e.g., access to the labor market and well-being) of marginalized ethnic groups (Motti-Stefanidi et al., 2014). Among Roma students, previous research has shown that engagement tends to decline significantly after the acquisition of basic literacy and numeracy skills (e.g., Rosário et al., 2017; Moreira et al., 2022a,b, 2023a,b), contributing to low attendance in secondary education and elevated dropout rates around the age of 16 (Organisation for Economic Co-operation and Development [OECD], 2022). Consistent with previous studies (e.g., Martin et al., 2022; Motti-Stefanidi et al., 2012, 2014), our findings reveal a progressive decline in emotional, behavioral, and cognitive engagement among Roma students in the 5th and 6th grades. This disengagement trajectory is particularly concerning given its association with early school leaving and limited educational attainment (Organisation for Economic Co-operation and Development [OECD], 2022).

Notably, the results underscore that school engagement among Roma students is highly context-dependent, shaped by the quality of relationships across school, family, and community domains.

Our findings demonstrated that students in this study showed greater involvement in informal and recreational school spaces, where intra-ethnic peer interactions were more prevalent, while simultaneously expressing negative emotions toward structured learning activities and showing reduced attendance in classrooms. These peer relationships, while fostering psychological well-being and a sense of belonging (Ladd et al., 1996; Liu et al., 2024; Wang and Eccles, 2012), may also inadvertently limit opportunities for inter-ethnic contact and engagement in formal academic settings. This pattern aligns with Méreiné-Berki et al. (2021), who argue that bonding social capital within Roma communities, though protective, can constrain the development of bridging ties with mainstream institutions, thereby reinforcing separation strategies over integrative ones (Moreira et al., 2022b, 2023a,b; Ward and Geeraert, 2016). In line, Berry’s (1997) acculturation framework posits that when integration is not supported by the host society, separation becomes a more viable strategy.

The observed decline in the perceived quality of teacher-student relationships further exacerbates this disengagement, fueling emotional barriers that hinder their engagement with formal learning. As highlighted by Makarova et al. (2023), the quality of these relationships is a critical determinant of school success, particularly for minoritized students. Supportive interactions with teachers and non-Roma peers are essential for facilitating positive acculturation processes and enhancing the perceived utility value of school (Bereményi and Carrasco, 2017; Teuscher and Makarova, 2018). Our findings suggest that disengagement among Roma students is a compounding process involving negative feelings about schoolwork (emotional disengagement), increased maladjustment in behaviors and expectations (behavioral disengagement), and poor self-regulation strategies (cognitive disengagement). As evidenced in the literature (e.g., Appleton et al., 2008), these dimensions are interrelated and mutually influence each other over time, underscoring the need for early and sustained intervention.

Furthermore, the observed engagement patterns with school space and disengagement with the classroom are closely tied to students’ perceptions of the value of education. In line with expectancy-value theory (Eccles and Wigfield, 2002), students from Roma groups often assign instrumental value to schooling, perceiving it as a tool to secure employment or avoid adverse outcomes (Moreira et al., 2022a,b, 2023a,b; Mendes et al., 2023). While these motivations may encourage attendance, students may benefit from the intrinsic task value needed for sustained interest and engagement in learning (e.g., Cai et al., 2023; Lee et al., 2016), especially when facing acculturation hassles (e.g., discrimination experiences, lack of cultural capital). The limited development of intrinsic value may hinder the formation of long-term academic goals (Durik et al., 2006), thereby constraining students’ educational aspirations and reinforcing short-term, pragmatic orientations (Kasser, 2016; Wigfield et al., 2015). For students from Roma groups, aspirations emerge from the complex navigation between cultural belonging and the demands of mainstream institutions (Berki et al., 2025; Méreiné-Berki et al., 2021; Dimitrova et al., 2018). As demonstrated in previous studies (e.g., Caetano et al., 2024; Obrovská et al., 2023), the structural features of schools and the quality of teacher-student relationships can significantly influence family engagement and students’ educational trajectories.

Altogether, the results of this study have important implications for educational policy and practice. The identified patterns of disengagement reflect structural and cultural tensions that disproportionately affect Roma students (Li et al., 2014; Wylie and Hodgen, 2012). The erosion of bonding capital without bridging and linking ties can undermine well-being and social mobility (Berki et al., 2025; Méreiné-Berki et al., 2021). Yet, underachievement among Roma students continues to be interpreted through a deficit-based lens that places responsibility on students and families, while neglecting the institutional role of schools in fostering engagement (e.g., Caetano et al., 2024). To address these challenges, our findings and previous literature (e.g., Salgado-Orellana et al., 2019) claim the need to promote intercultural education in schools (e.g., teacher training in intercultural education, involving cultural mediators) to create spaces where Roma students can thrive academically and socially.

### Limitations and further research

This study benefits from a culturally sensitive and longitudinal qualitative design, allowing for a nuanced understanding of school engagement trajectories among Roma students. The use of two interview waves enabled the capture of developmental changes and reflections over time, while the involvement of researchers familiar with Roma communities helped foster trust and minimize cultural misunderstandings. However, the study also presents limitations.

First, findings rely on a single data source and data collection method. Although rich in depth, it limits triangulation and makes it challenging to learn the influence of context-related mechanisms on (dis)engagement trajectories of students from Roma backgrounds. Further multi-source data and longitudinal research are needed to examine the school (dis)engagement trajectories and educational beliefs and trace the directions of effects between context-related factors and school engagement over time. Moreover, future quantitative studies could consider approaching students’ engagement trajectories in learning, as current data provide evidence that engagement patterns are different in school and learning.

Second, although all participants were proficient in Portuguese, the open-ended nature of the questions and the cognitive demands of reflection may have posed challenges for some students, particularly those with learning difficulties or limited expressive skills. While interviewers made efforts to ensure comprehension and adapted language accordingly, this may have influenced the depth and accuracy of responses.

Third, the small sample in this study limits generalizability and the ability to compare across Roma subgroups systematically. Therefore, findings are not necessarily illustrative of other groups, even within the Roma community or educational contexts. Future studies with larger and more balanced samples could explore how subgroup affiliation intersects with engagement trajectories, educational beliefs, and acculturation experiences.

Fourth, researcher positionality may have shaped both data collection and interpretation. Although researchers’ familiarity with Roma communities facilitated rapport and cultural understanding, differences in ethnicity, educational background, and institutional affiliation may still have influenced participants’ responses. From a post-positivist perspective, we acknowledge that complete objectivity is unattainable and therefore implement strategies to minimize bias, including the use of semi-structured interviews, reflexive practices, and peer debriefing with colleagues not involved in data collection. We also prioritized participants’ autonomy by encouraging clarification or correction of interpretations during the interviews. Future work would benefit from incorporating collaborative analysis with community members to further enhance cultural relevance and validity.

## Conclusion and implications

Despite the educational investments in Roma education, the overall impact on improving the school outcomes of students from Roma backgrounds has been limited. This study makes important theoretical contributions while contributing to the growing body of research that emphasizes the centrality of school engagement in shaping positive educational trajectories and long-term adaptation, including labor market integration (Dotterer and Lowe, 2011; Rosário et al., 2016). Moreover, this work underscores the importance of considering cultural and ecological factors in student engagement and motivation theories (Roundfield et al., 2018). Examining engagement trajectories among 5th and 6th-grade students from Roma backgrounds reveals a nuanced pattern of (dis)engagement that operates across multiple dimensions of school life. While experiencing a positive engagement trend with non-curricular activities (e.g., playing with friends in the yard and going to the cafeteria), these students exhibit significant symptoms of disengagement with the learning process. This disengagement is compounded by a reliance on intra-ethnic peer networks for social support and belonging, with limited evidence of positive relationships with non-Roma peers or teachers. Such patterns suggest that Roma students may experience school as a socially fragmented space, where cultural dissonance and perceived exclusion hinder their academic integration. These findings underscore the critical need to address both the structural and relational barriers that constrain the engagement of these students. Factors such as systemic discrimination, lack of intercultural competence among educators, and a misalignment between students’ needs and institutional responses contribute to a school climate that may inadvertently reinforce marginalization. In this context, disengagement from mainstream educational culture may be a protective strategy, reinforcing ethnic identity while limiting access to broader academic and social opportunities. To counter these dynamics, educational policies and practices should actively address the academic and social needs of students in Roma groups. To counter these dynamics, educational policies and practices should address the academic and social needs of students in Roma groups. Based on the study’s results and previous studies (e.g., Mendes et al., 2023; Obrovská et al., 2023), targeted school interventions are needed to facilitate positive inter-ethnic interactions and build supportive relationships with teachers and non-Roma peers. As evidenced in prior studies (e.g., Nishina et al., 2019), peer-mentoring programs have demonstrated effectiveness in enhancing academic motivation, social integration, and intergroup understanding among minoritized students. These programs can also serve as platforms for fulfilling students’ basic psychological needs (e.g., by creating an environment where Roma students feel included and valued), promoting school engagement.

Moreover, early and sustained interventions may be needed to help these students develop an intrinsic value of learning and education, preventing hidden disengagement and dropout. Given the crucial role of teachers in supporting students’ developmental tasks (i.e., aspirations, school engagement, and achievement), especially for minoritized students, educators would benefit from professional development in culturally responsive teaching and practices. Caetano et al. (2024) highlight that such training can enhance educators’ capacity to create inclusive learning environments, mitigate implicit bias, and affirm the cultural identities of the Roma community. Other culturally responsive interventions such as the integration of Roma cultural mediators to bridge communication between schools and families, the adaptation of curricular content to reflect Roma students lived experiences and cultural references, and the creation of structured spaces for dialog and collaboration between Roma families and school staff could aim to reduce cultural dissonance and foster a sense of belonging within the school environment. Finally, interventions should focus on developing critical psychological resources, including hope and self-regulated learning strategies (Núñez et al., 2013). These skills are essential for helping Roma students navigate acculturation-related stressors and adapt to the demands of the school context.

## Data Availability

The datasets presented in this article are not readily available because interviews contain information that could compromise the privacy of research participants. Requests to access the datasets should be directed to taniatmoreira@gmail.com.

## References

[ref1] Appleton J. J. Christenson S. L. Furlong M. J. (2008). Student engagement with school: critical conceptual and methodological issues of the construct. Psychol. Sch. 45, 369–386. doi: 10.1002/pits.20303

[ref2] Azevedo R. Rosário P. Núñez J. C. Vallejo G. Fuentes S. Magalhães P. (2023). A school-based intervention on elementary students’ school engagement. Contemp. Educ. Psychol. 73:102148. doi: 10.1016/j.cedpsych.2023.102148, PMID: 40757858

[ref3] Bereményi B. Á. Carrasco S. (2017). “Bittersweet success: the impact of academic achievement among the Spanish Roma after a decade of Roma inclusion” in Second international handbook of urban education. eds. Pink W. Noblit G. (Cham: Springer International Handbooks of Education. Springer), 1169–1198.

[ref4] Berki B. Málovics G. Creţan R. (2025). Aspirations versus reality: factors influencing the vertical social mobility of urban Roma mothers living in extreme poverty. Habitat Int. 158:103334. doi: 10.1016/j.habitatint.2025.103334

[ref5] Berry J. W. (1997). Immigration, acculturation, and adaptation. Appl. Psychol. 46, 5–34. doi: 10.1111/j.1464-0597.1997.tb01087.x, PMID: 40757858

[ref6] Berry J. W. (2005). Acculturation: living successfully in two cultures. Int. J. Intercult. Relat. 29, 697–712. doi: 10.1016/j.ijintrel.2005.07.013

[ref8] Braun V. Clarke V. (2012). “Thematic analysis” in APA, Handbook of research methods in psychology, 2: Research designs: Quantitative, qualitative, neuropsychological, and biological. (eds.) Cooper H Camic PM Long DL Panter AT Rindskopf D Sher KJ. (American Psychological Association), 57–71. doi: 10.1037/13620-004

[ref9] Caetano P. Mendes M. M. Pinheiro S. Mourão S. Candeias P. Magano O. (2024). Teachers’ perspectives on determinants of educational (under)achievement of Cigano/Roma students in Portugal. Intercult. Educ. 35, 517–538. doi: 10.1080/14675986.2024.2403048

[ref10] Cai Y. King R. B. McInerney D. M. (2023). The concurrent trajectories of utility value, metacognitive strategy use, and achievement. J. Exp. Educ. 91, 472–493. doi: 10.1080/00220973.2022.2053496

[ref12] Chase P. A. Hilliard L. J. Geldhof G. J. Warren D. J. Lerner R. M. (2014). Academic achievement in the high school years: the changing role of school engagement. J. Youth Adolesc. 43, 884–896. doi: 10.1007/s10964-013-0085-4, PMID: 24477498

[ref14] Cooper M. Rodgers B. (2006). Proposed scoring scheme for qualitive thematic analysis: University of Strathclyde.

[ref15] Council of Europe. (2020a) Roma and Traveller inclusion. Available online at: https://rm.coe.int/09000016809f2a53. (Accessed September 19, 2023).

[ref16] Council of Europe. (2020b). Strategic action plan for Roma and Traveller inclusion (2020–2025). Available online at: https://edoc.coe.int/en/roma-and-travellers/8508-council-of-europe-strategic-action-plan-for-roma-and-traveller-inclusion-2020-2025.html (Accessed September 19, 2023)

[ref9003] Dimitrova R. Ferrer-Wreder L. Ahlen J. (2018). School climate, academic achievement and educational aspirations in Roma minority and Bulgarian majority adolescents. Child & Youth Care Forum, 47, 645–658. doi: 10.1007/s10566-018-9451-4 30237688 PMC6133028

[ref17] Direção-Geral de Estatísticas da Educação e Ciência [DGEEC] (2019) Perfil escolar das Comunidades Ciganas. Available online at: http://www.dgeec.mec.pt/np4/1136.html. (Accessed July 24, 2023).

[ref18] Dotterer A. M. Lowe K. (2011). Classroom context, school engagement, and academic achievement in early adolescence. J. Youth Adolesc. 40, 1649–1660. doi: 10.1007/s10964-011-9647-5, PMID: 21400208

[ref19] Durik A. M. Vida M. Eccles J. S. (2006). Task values and ability beliefs as predictors of high school literacy choices: a developmental analysis. J. Educ. Psychol. 98, 382–393. doi: 10.1037/0022-0663.98.2.382

[ref21] Eccles J. S. Wigfield A. (2002). Motivational beliefs, values, and goals. Annu. Rev. Psychol. 53, 109–132. doi: 10.1146/annurev.psych.53.100901.135153, PMID: 11752481

[ref23] European Union Agency for Fundamental Rights (FRA) (2021) Roma in 10 countries: Main results. Available online at: https://fra.europa.eu/en/publication/2022/roma-survey-findings. (Accessed September 19, 2023).

[ref24] Fall A. Roberts G. (2012). High school dropouts: interactions between social context, self-perceptions, school engagement, and student dropout. J. Adolesc. 35, 787–798. doi: 10.1016/j.adolescence.2011.11.004, PMID: 22153483 PMC3478125

[ref25] Frazer H. Marlier E. (2011) Promoting the social inclusion of Roma. Available online at: https://liser.elsevierpure.com/fr/publications/promoting-the-social-inclusion-of-roma. (Accessed September 19, 2023).

[ref26] Fredricks J. A. Blumenfeld P. C. Paris A. H. (2004). School engagement: potential of the concept, state of the evidence. Rev. Educ. Res. 74, 59–109. doi: 10.3102/00346543074001059

[ref27] Fredricks J. A. Filsecker M. Lawson M. A. (2016). Student engagement, context, and adjustment: addressing definitional, measurement, and methodological issues. Learn. Instr. 43, 1–4. doi: 10.1016/j.learninstruc.2016.02.002

[ref28] Fredricks J. A. Ye F. Wang M.-T. Brauer S. (2019). “Profiles of school disengagement: not all disengaged students are alike” in Handbook of student engagement interventions: Working with disengaged students. eds. Fredricks J. A. Reschly A. L. Christenson S. L. (Elsevier Academic Press), 31–43. doi: 10.1016/B978-0-12-813413-9.00003-6

[ref29] Guo J. Parker P. D. Marsh H. W. Morin A. J. S. (2015). Achievement, motivation, and educational choices: a longitudinal study of expectancy and value using a multiplicative perspective. Dev. Psychol. 51, 1163–1176. doi: 10.1037/a0039440, PMID: 26053150

[ref30] Hamilton P. (2018). Engaging gypsy and Traveller pupils in secondary education in Wales: tensions and dilemmas of addressing difference. Int. Stud. Sociol. Educ. 27, 4–22. doi: 10.1080/09620214.2017.1377100

[ref31] Hancock K. J. Zubrick S. R. (2015). Children and young people at risk of disengagement from school. Commissioner for Children and Young People Western Australia. Available online at: https://www.researchgate.net/profile/Kirsten-Hancock-2/publication/281257513_Children_and_young_people_at_risk_of_disengagement_from_school_literature_review/links/55dd3b4108ae83e420ee62bc/Children-and-young-people-at-risk-of-disengagement-from-school-literature-review.pdf. (Accessed September 19, 2023).

[ref32] Harackiewicz J. M. Smith J. L. Priniski S. J. (2016). Interest matters: the importance of promoting interest in education. Policy Insights Behav. Brain Sci. 3, 220–227. doi: 10.1177/2372732216655542, PMID: 29520371 PMC5839644

[ref33] Hirschfield P. J. Gasper J. (2010). The relationship between school engagement and delinquency in late childhood and early adolescence. J. Youth Adolesc. 40, 3–22. doi: 10.1007/s10964-010-9579-5, PMID: 20706779

[ref34] Kasser T. (2016). Materialistic values and goals. Annu. Rev. Psychol. 67, 489–514. doi: 10.1146/annurev-psych-122414-033344, PMID: 26273896

[ref35] Kraus P. A. (2012). The politics of complex diversity: a European perspective. Ethnicities 12, 3–25. doi: 10.1177/1468796811426952

[ref36] Ladd G. W. Kochenderfer B. J. Coleman C. C. (1996). Friendship quality as a predictor of young children’s early school adjustment. Child Dev. 67, 1103–1118. doi: 10.2307/1131882, PMID: 8706512

[ref37] Lauritzen S. M. Nodeland T. S. (2018). “What is the problem represented to be?” two decades of research on Roma and education in Europe. Educ. Res. Rev. 24, 148–169. doi: 10.1016/j.edurev.2018.04.002

[ref38] Lee H. Fawcett J. DeMarco R. (2016). Storytelling/narrative theory to address health communication with minority populations. Appl. Nurs. Res. 30, 58–60. doi: 10.1016/j.apnr.2015.09.004, PMID: 27091254

[ref39] Li Y. Agans J. P. Chase P. A. Arbeit M. R. Weiner M. B. Lerner R. M. (2014). School engagement and positive youth development: a relational developmental systems perspective. Teach. Coll. Rec. 116, 37–57. doi: 10.1177/016146811411601303

[ref40] Li Y. Lerner R. M. (2011). Trajectories of school engagement during adolescence: implications for grades, depression, delinquency, and substance use. Dev. Psychol. 47, 233–247. doi: 10.1037/a0021307, PMID: 21244162

[ref41] Liu J. Zhu Z. Kong X. Coplan R. J. Zhao K. Li D.. (2024). Developmental trajectories of emotional school engagement from middle to late childhood in mainland China: contributions of early peer relationships and academic achievement. Eur. J. Psychol. Educ. 39, 151–170. doi: 10.1007/s10212-023-00691-8

[ref42] Lyberg L. Stange M. Harkness J. Mohler P. Pennell B.-E. Japec L. (2014). “A review of quality issues associated with studying hard-to-survey populations” in Hard-to-survey populations. eds. Edwards B. Wolter K. M. Bates N. Tourangeau R. Johnson T. P. (Cambridge University Press), 82–108. doi: 10.1017/CBO9781139381635

[ref43] Makarova E. (2019). Acculturation and school adjustment of minority students: school and family-related factors. Intercult. Educ. 30, 445–447. doi: 10.1080/14675986.2019.1643559

[ref44] Makarova E. Birman D. (2015). Cultural transition and academic achievement of students from ethnic minority backgrounds: a content analysis of empirical research on acculturation. Educ. Res. 57, 305–330. doi: 10.1080/00131881.2015.1058099

[ref45] Makarova E. Döring A. K. Auer P. ‘t Gilde J. Birman D. (2023). School adjustment of ethnic minority youth: a qualitative and quantitative research synthesis of family-related risk and resource factors. Educ. Rev. 75, 324–347. doi: 10.1080/00131911.2021.1905610

[ref46] Martin A. J. Burns E. C. Collie R. J. Cutmore M. MacLeod S. Donlevy V. (2022). The role of engagement in immigrant students’ academic resilience. Learn. Instr. 82:101650. doi: 10.1016/j.learninstruc.2022.101650, PMID: 40757858

[ref47] Mendes M. M. Magano O. (2021). Educational situation of Portuguese Ciganos: social changes versus social continuities. (Mendes M. M. Magano O. Toma S. Eds.), Social and economic vulnerability of Roma people: Key factors for the success and continuity of schooling levels (19–38). Springer International Publishing. doi: 10.1007/978-3-030-52588-0_2

[ref48] Mendes M. M. Magano O. Mourão S. Pinheiro S. (2023). In-between identities and hope in the future: experiences and trajectories of Cigano secondary students. Comp. J. Comp. Int. Educ. 54, 479–498. doi: 10.1080/03057925.2023.2170170

[ref49] Méreiné-Berki B. Málovics G. Creţan R. (2021). “You become one with the place”: social mixing, social capital, and the lived experience of urban desegregation in the Roma community. Cities 117:103302. doi: 10.1016/j.cities.2021.103302, PMID: 40757858

[ref50] Moreira T. Martins J. Núñez J. C. Oliveira A. Martins J. Rosário P. (2023a). Acculturation and school engagement: the case of Portuguese students with Roma background. Rev. Psicol. 28, 67–79. doi: 10.1016/j.psicod.2022.11.003

[ref51] Moreira T. Martins J. Silva C. Berrocal de Luna E. Martins J. Moreira D.. (2023b). Building partnerships in education through a story-tool based intervention: parental involvement experiences among families with Roma backgrounds. Front. Psychol. 14. doi: 10.3389/fpsyg.2023.1012568, PMID: 36968727 PMC10033949

[ref52] Moreira T. Rosário P. Azevedo R. Núñez J. C. Fuentes S. (2022b). Living on a double-edged sword: intergenerational perspectives of women from gypsy groups about the influence of education on cultural identity. Int. J. Educ. Res. 111:101915. doi: 10.1016/j.ijer.2021.101915, PMID: 40757858

[ref53] Moreira T. Rosário P. Martins J. Silva C. Moreira D. Ribeiro L. (2022a). ‘Caught between dreams and deeds’: the hope avenue for school success of students with a Roma background. In Dutton J. (Dutton J. Ed.), The psychology of self-regulation (pp. 147–175). Nova Science Publishers, Inc.

[ref54] Motti-Stefanidi F. (2018). Resilience among immigrant youth: the role of culture, development and acculturation. Dev. Rev. 50, 99–109. doi: 10.1016/j.dr.2018.04.002

[ref55] Motti-Stefanidi F. (2023). Acculturation and resilience of immigrant-origin youth: do their school experiences reflect nonimmigrants’ “native supremacy”? Dev. Psychopathol. 35, 2155–2167. doi: 10.1017/S0954579423000895, PMID: 37539699

[ref56] Motti-Stefanidi F. Asendorpf J. B. Masten A. S. (2012). The adaptation and well-being of adolescent immigrants in Greek schools: a multilevel, longitudinal study of risks and resources. Dev. Psychopathol. 24, 451–473. doi: 10.1017/s0954579412000090, PMID: 22559124

[ref57] Motti-Stefanidi F. Masten A. Asendorpf J. B. (2014). School engagement trajectories of immigrant youth. Int. J. Behav. Dev. 39, 32–42. doi: 10.1177/0165025414533428

[ref59] Nishina A. Lewis J. A. Bellmore A. Witkow M. R. (2019). Ethnic diversity and inclusive school environments. Educ. Psychol. 54, 306–321. doi: 10.1080/00461520.2019.1633923

[ref60] Núñez J. Rosário P. Vallejo G. González-Pienda J. (2013). A longitudinal assessment of the effectiveness of a school-based mentoring program in middle school. Contemp. Educ. Psychol. 38, 11–21. doi: 10.1016/j.cedpsych.2012.10.002

[ref61] Obrovská J. Aguiar C. Silva C. S. Petrogiannis K. (2023). Predictors of educational aspirations of Roma mothers in Czech Republic, Greece, and Portugal. Soc. Psychol. Educ. 26, 1063–1088. doi: 10.1007/s11218-023-09780-4

[ref62] Organisation for Economic Co-operation and Development [OECD]. (2022). Review of inclusive education in Portugal. Organisation for Economic Co-operation and Development. Available online at: https://www.oecd-ilibrary.org/education/review-of-inclusive-education-in-portugal_a9c95902-en (Accessed August 26, 2023).

[ref64] Robinson O. C. (2014). Sampling in interview-based qualitative research: a theoretical and practical guide. Qual. Res. Psychol. 11, 25–41. doi: 10.1080/14780887.2013.801543

[ref65] Rosário P. Núñez J. C. Ferrando P. J. Paiva M. O. Lourenço A. Cerezo R.. (2013a). The relationship between approaches to teaching and approaches to studying: a two-level structural equation model for biology achievement in high school. Metacogn. Learn. 8, 47–77. doi: 10.1007/s11409-013-9095-6

[ref66] Rosário P. Núñez J. C. Valle A. Paiva O. Polydoro S. A. J. (2013b). Approaches to teaching in high school when considering contextual variables and teacher variables. Rev. Psicol. 18, 25–45. doi: 10.1387/RevPsicodidact.6215

[ref67] Rosário P. Núñez J. C. Vallejo G. Azevedo R. Pereira R. Moreira T.. (2017). Promoting gypsy children’s behavioural engagement and school success: evidence from a four-wave longitudinal study. Br. Educ. Res. J. 43, 554–571. doi: 10.1002/berj.3271

[ref68] Rosário P. Núñez J. C. Vallejo G. Cunha J. Azevedo R. Pereira R.. (2016). Promoting gypsy children school engagement: a story-tool project to enhance self-regulated learning. Contemp. Educ. Psychol. 47, 84–94. doi: 10.1016/j.cedpsych.2015.11.005

[ref69] Rosário P. Pereira B. Magalhães P. Moreira T. Mesquita S. Fuentes S.. (2020). A brief school-based intervention on gypsy culture: a longitudinal cluster randomized trial. J. Educ. Res. 113, 462–474. doi: 10.1080/00220671.2020.1855096

[ref70] Roundfield K. D. Sánchez B. McMahon S. D. (2018). An ecological analysis of school engagement among urban, low-income Latino adolescents. Youth Soc. 50, 905–925. doi: 10.1177/0044118X16639986

[ref71] Rutigliano A. (2020). Inclusion of Roma students in Europe: a literature review and examples of policy initiatives. (OECD Education Working Papers No. 228). OECD Education Working Papers.

[ref72] Salgado-Orellana N. Berrocal de Luna E. Sánchez-Núñez C. A. (2019). Intercultural education for sustainability in the educational interventions targeting the Roma student: a systematic review. Sustainability 11. doi: 10.3390/su11123238, PMID: 40607831

[ref73] Schachner M. K. He J. Heizmann B. Van de Vijver F. J. R. (2017). Acculturation and school adjustment of immigrant youth in six European countries: findings from the programme for international student assessment (PISA). Front. Psychol. 8:649. doi: 10.3389/fpsyg.2017.00649 28522980 PMC5415604

[ref74] Smith A. R. (2017). Insights into the shifting perspectives of members of the gypsy and Traveller community on schooling, and implications for school leaders. Manag. Educ. 31, 14–20. doi: 10.1177/0892020616683337

[ref75] Stenroos M. Helakorpi J. (2021). “The multiple stories in Finnish Roma schooling” in Social and economic vulnerability of Roma people: Key factors for the success and continuity of schooling levels. eds. Mendes M. M. Magano O. Toma S. (Springer), 99–116. doi: 10.1007/978-3-030-52588-0_7

[ref76] Teuscher S. Makarova E. (2018). Students’ school engagement and their truant behavior: do relationships with classmates and teachers matter? J. Educ. Learn. 7, 124–137. doi: 10.5539/jel.v7n6p124

[ref9001] Wang M.‐T. Degol J. (2014). Staying engaged: Knowledge and research needs in student engagement.Child Development Perspectives, 8, 137–143. doi: 10.1111/cdep.12073 27087833 PMC4833401

[ref77] Wang M.-T. Eccles J. S. (2012). Social support matters: longitudinal effects of social support on three dimensions of school engagement from middle to high school. Child Dev. 83, 877–895. doi: 10.1111/j.1467-8624.2012.01745.x, PMID: 22506836

[ref78] Wang M.-T. Fredricks J. A. (2013). The reciprocal links between school engagement, youth problem behaviors, and school dropout during adolescence. Child Dev. 85, 722–737. doi: 10.1111/cdev.12138, PMID: 23895361 PMC3815520

[ref79] Wang M.-T. Fredricks J. Ye F. Hofkens T. Linn J. S. (2019). Conceptualization and assessment of adolescents’ engagement and disengagement in school: a multidimensional school engagement scale. Eur. J. Psychol. Assess. 35, 592–606. doi: 10.1027/1015-5759/a000431

[ref80] Ward C. Geeraert N. (2016). Advancing acculturation theory and research: the acculturation process in its ecological context. Curr. Opin. Psychol. 8, 98–104. doi: 10.1016/j.copsyc.2015.09.021, PMID: 29506811

[ref81] Wigfield A. Eccles J. S. Fredricks J. A. Simpkins S. Roeser R. W. Schiefele U. (2015). “Development of achievement motivation and engagement” in Handbook of child psychology and developmental science: Socioemotional processes. eds. Lamb M. E. Lerner R. M.. 7th ed (John Wiley & Sons, Inc.), 657–700. doi: 10.1002/9781118963418.childpsy316

[ref82] Wilding D. The educational experiences of gypsy Travellers: the impact of cultural dissonance. Reinvention: A Journal of Undergraduate Research, (2008). 1, 1–12. Available online at: https://warwick.ac.uk/fac/cross_fac/iatl/reinvention/archive/volume1issue1/wilding.

[ref83] Wylie C. Hodgen E. (2012). “Trajectories and patterns of student engagement: evidence from a longitudinal study” in Handbook of research on student engagement. eds. Christenson S. L. Reschly A. L. Wylie C. (Springer Science + Business Media), 585–599. doi: 10.1007/978-1-4614-2018-7_28

[ref84] Želinský T. Gorard S. Siddiqui N. (2021). Increasing understanding of the aspirations and expectations of Roma students. Br. J. Sociol. Educ. 42, 588–606. doi: 10.1080/01425692.2021.1872366

